# Typhoid Pneumonia—Its Treatment, Etc.

**Published:** 1879-02-20

**Authors:** Alban S. Payne

**Affiliations:** Virginia


### Southern Medical Record.

EDITORS:

T. S. Powell, M.D. W. T. Goldsmith, M.D. R. C. Word, M.D.

*R. C. WORD, M.D., Managing Editor.*

☞All Communications and Letters on Business connected with the Record must be addressed to the Managing Editor.

Vol. IX. ATLANTA, GA., FEB. 20, 1879. No. 2.

### ORIGINAL AND SELECTED ARTICLES.

### *TYPHOID PNEUMONIA—ITS TREATMENT, ETC.*

RY ALBAN S. PAYNE, M. D., OF VIRGINIA.

In searching for the rationale of vital actions or phenomena in disease as well as in health, we believe with those who deem it necessary to begin with the nervous system, the only parts of the body, within our knowledge, which originate, carry forward and control action. Nerves first affected, then necessarily follows the affection of the vital actions or functions dependent on nerves. In other words, it is our opinion that the poison of miasmata acts primarily on the nerves, and through them secondarily on the sanguiferous system, producing a blood poison.

The first visible change in inflamed parts is allowed by all to be relaxation of the capillaries of the part inflamed, but there must be previously to this relaxation, an injury to the nerves supplying these vessels, for when there is a loss of action (tonic contractions), there must be a loss of nervous power, and in cases of inflammation not ending in resolution, the loss of nervous power is so great that disorganization ensues, followed by suppuration or death of the whole of the part inflamed. In fevers we have the same phenomena, but the capillary relaxation is general, and less in degree. We trust that every practitioner at this day is informed of the existence of two almost independently acting systems of nerves, called the "cerebro-spinal" and the "ganglionic," or "organic nerves"; the former, capable of thought, sensation and voluntary motion; and the latter, presiding over the functions of organic life. All the functions of the organism are performed through the agency of the capillaries; hence the par vagum, or great sympathetic, is the nerve of the capillaries, and hence it is this nerve which is primarily injured in all inflammations and fevers. And we believe that in all sthenic inflammations and inflammatory idiopathic fevers it is this nerve alone; whilst asthenic or typhoid diseases of every grade have superadded a similar affection of the cerebro-spinal system. We also believe that irritation is often charged to the door of inflammation, and whilst venesection is beneficial in inflammation, it is equally prejudicial in irritations.

Dr. Hasbrouch very sensibly says: "As a general fact, inflammation of serous tissues excites more than inflammation of the cellular or parenchymatous structures, and inflammation of these more than that of the mucous. Acute inflammation of the ligaments and periosteum often causes intense general excitement. As a general fact, too, cases of the highest excitement admit safely of a higher grade of vascular action than those in which it is more moderate; or in other words, the force of the heart's action, judged by the pulse, will need to be reduced lower in inflammations of some tissues and organs than in others. A pulse, for instance, which might be allowable in pleuritis would demand bleeding in pneumonia, or bronchitis, and decidedly so in muco-enteritis, or dysentery. We would reduce the pulse lower in inflammation of the brain than in that of the lungs, or liver."

But why this difference, if bleeding be practiced solely to bring within a certain limit the heart's vis a tergo? Why will a certain force in the heart inject seriously the capillaries of one organ and not of another—of the mucous tissue and not of the serous? We can explain the fact in no other way than by supposing that some organs and tissues are naturally more lax, and the capillaries more easily distended than they are in others.

We have often heard teachers of medicine and others speak of bleeding in connection with puerperal peritonitis, phrenitis, pericarditis (when it could be ascertained to exist) and iritis, as if alone it could arrest the inflammation, and the more blood was drawn the surer was the cure. It was not understood that we can bleed our patients into, as well as out of inflammations, and even apoplexies. Supposing we have a case of acute meningitis, idiopathic, or following an injury, with the unusually accompanying high vascular excitement, and we bleed and bleed again, until the pulse sinks down to or below the standard of health, and the inflammation be unsubdued. We apply cold to the head, or blisters to the neck. The bowels move freely, and the patient takes antimony, but still the pain, or delirium, the heat, intolerance of light and noise, are the same. Here is a vital organ diseased; must we bleed again? If we do we shall increase the inflammatory congestion, by adding a general relaxation to a local one, and thus kill our patient.

We said it was not understood that we could bleed patients into as well as out of inflammation, or that the opposite states of repletion and depletion tended to the same results. There is no fact in medicine better established now; and so nearly do the symptoms of exhaustion simulate those of sthenia, that it requires great care and sound discriminating judgment to make out the true diagnosis. We are sure that nothing in our experience has been more difficult than to determine, in some cases of fever following confinement, where there has been severe abdominal pain, and tenderness on pressure, or a violent headache with ringing of the ears, flushed face, etc., whether these symptoms were caused by a veritable acute peritonitis, or phrenitis, demanding, of course, venesection; or by morbid sensibility of the nerves, the result of too great loss of blood, and requiring opium and tonic. Cases are not unfrequently met with of acute sthenic inflammation, with a small and feeble pulse, in which bleeding is quite as necessary as it is in the more ordinary forms, which are attended with great excitement and a bounding pulse. A person, for instance, is seized in the midst of apparent health with a chill or chilliness followed by headache, pain in the back and limbs, irregular heat of surface, a sense of oppression and debility, and an apparently feeble and frequent pulse. He may have pneumonia, without pain or cough, or he may have inflammation of some other part, without the usual symptoms which are dependent upon the nerves of sensation. This is a case of inflammation (injury to the nervous system and subsequent capillary relaxation), which, in its origin stands more nearly related to some general constitutional peculiarity or depravity of nervous function, than ordinary acute inflammations do; and which, in its sympathetic effects upon the general nervous apparatus, either through the previously depraved condition of the general functions, or from the local force this constituttonal condition gives it, overwhelms innervation to an extent that produces general inaction and consequent congestion. Bleeding is called for in this state of the system, but it is not evidenced by the pulse and excitement, but by the circumstances of the attack. And it is called for, not to prevent the heart's increased action from keeping up a distension of the capillaries of the inflamed part, for no such increased action exists, but to unload the whole vascular system, even the heart itself. Extensive, or intense inflammation of vital organs will also sometimes produce this depressed or overwhelmed condition of the constitution. Allowing, then, for all these modifications of sthenic excitement, and for the occasional exceptional cases of depression occurring in some peculiar conditions of the general system, or in some severe inflammations of vital organs, it will be proper to say that the pulse is the guide in the use of the lancet. The force of the pulse reduced to a point which tells that the heart's unnatural forcing power upon the capillaries is removed, and we will have accomplished all that bleeding ever can accomplish. But in asthenic diseases we should recollect we have an entirely different state of things.

We have already stated, that in our view, diseases of the sthenic or inflammatory type have their origin in lesion of the organic system of nerves alone, whilst those of the asthenic or typhoid type have superadded a similar lesion of the cerebro-spinal system. If the lesion be local at first, it is propagated by sympathy; if general, as in fevers, it is produced by immediate impression. Let us see how the symptoms will bear us out in this opinion. In sthenic diseases we have anorexia, white tongue, torpid bowels, hot skin, scanty urine, etc., all evidences of impaired functions of the organic system, whilst thought, sensation and voluntary motion, functions of the cerebro-spinal nerves, are comparatively unaffected. High excitement (exalted action, the opposite of that paralyzed condition which destroys functions and originates disease) may cause delirium and even convulsions; but ordinarily the eye has the expression of health (perhaps a little brightened by the excitement), the perceptions are clear, the muscular strength unabated, and the heart beats with more than its usual force. In asthenia, although we have an increase of the derangement of the secretions, the prominent diagnostics of this condition consists in the heavy eye, oppressed intellect, subdued muscular power and feeble pulse; and closely connected with these is a deteriorated state of the blood.

"At different times in the history of medicine this fact has given rise to the humoral pathology, many of the symptoms of typhus and of some chronic diseases, seeming to be inexplicable, without taking into consideration the attendant deteriorated condition of the circulating fluids."

But blood cannot become deteriorated without a previous failure of the functions of the nerves which preside over its formation. What part or parts of the body perform this function? Where is the food converted? And what converts it into blood? Professor Meigs says: "In the heart and blood vessels, and by their lining membane." The heart is a muscular organ of great power, and most anatomists and physiologists teach that the arteries have a muscular coat, and hence both must be supplied with motor nerves. We know that the heart is thus supplied. And always when this supply is greatest in any organ, the organic nerves seem more dependent upon them than on other parts for their proper capabilities. In paralysis, nutrition is deficient and the muscles waste. This, then, is the explanation of the typhoid diathesis of the essential nature of typhus. Lesions of the cerebrospinal system, added to a similar lesion of the ganglionic, and embracing that important function of the system, the generation of blood. And that this function is deranged, whether situated in the endangium, as Professor Meigs terms it, or not, is proved, not only by the known impure state of the blood in these diseases, but by the inability of the system to supply promptly any loss that may be produced spontaneously or by venesection. The system in health has always a superabundance of adipose tissues from which support is drawn, in case the digestion of food becomes impaired, as in all acute affections. Hence in acute inflammatory affections, we bleed, and repeat it again and again, and still find a new supply; but emaciation progresses rapidly. Not so in typhoid: the least blood abstracted seems to be so much lost; nor can we increase the supply by stimulation; for blood cannot be made, or but slowly, and emaciation progresses equally slowly. It is often scarcely observable until convalescence commences, or until after protracted illness. Now, a priori, we should suppose that diseases which cut off the supply of blood to the system, would not be those to be relieved by the abstraction of any portion of it, and upon which the system has solely to depend. And this is a fact proved by all experience. Cases occasionally occur which simulate those of oppression in sthenia already mentioned, where in the premonitory stage the quantity of blood has not been reduced to the diminished demand for it, and where, by its excess, life is immediately endangered by congestion which will be benefitted by a cautious bleeding. In the progress of the disease too, if it be fever, a superimposed inflammation of some vital organ may, by its congestive or overwhelming effects upon the nervous system, produce the same effect, and the system has enough of the vital current left to admit of a topical abstraction. But these are exceptions, and the general fact remains, that typhoid diseases will not tolerate bleeding. Removing the injecting force of the heart (if it exists) will not relieve the capillaries, because the source of their power, the whole nervous system, is almost paralized, and left so by a corrupted condition of the blood, the result of disease of the blood-making function. But the excited state of the circulation in these diseases is without strength. The pulse is always compressible, which shows that the heart's power is diminished rather than increased as it is in asthenia, and that it cannot exert any injurious propulsive force. That the heart is weakened, and that this weakness is connected with some affection of the organ, and that the state of the blood corresponds with the affection, is proved by the heart's softened condition in extreme cases. Dr. Latham says: "There is softening of the heart which belongs to fevers of a typhoid type, when the mass of blood becomes loose and black, and uncoagulable, as if some foreign ingredient or some force had operated on it so as to change its chemical affinities, and destroy for the time its natural properties as blood."

Now do not these facts go far to prove that the making of blood is in some important manner connected with the heart? We think they do. And this admitted, we think that there can be no doubt of the correctness of our views of the *essential* nature of typhoid diseases. It is possible that the portion of the organic nerves which supplies the heart and its lining membranes, as also that of the arteries, and which, as we assume, presides over the blood-making function, should be primarily affected, and that the affection of the rest of the organic system, and the cerebro-spinal nerves also, should be secondary, as the result of impoverished or deteriorated blood. But how, if this be the case, can we account for the comparative immunity of the cerebro-spinal system in sthenic diseases in which all the secretions are deranged, implying an affection of the whole ganglionic system, and which should take in the lining of the heart as well as that of the stomach and intestines; unless the capillaries of the former hold that more intimate functional relation with the cerebro-spinal system, which we suppose they do.

And admitting this relation as the cause of this immunity in sthenia, brings us back to the cerebro-spinal lesion as the primary step in asthenia.

The mechanism of the human frame is too wonderfully, too fearfully made, to run one hour, much less for three score years and ten, by guess work or by chance. This being so, I hold that a certain given quantity of blood must be thrown through the brain and its meninges by the heart in a certain given number of seconds, and any departure from this is necessarily a departure from a physiological state to a pathological condition, and is always evidenced by a certain train of *prodromata*. The patent fact being ignored, that *too little* blood circulating through the brain and its appendage in a given number of seconds, as surely produces delirium as does too much blood circulating through the same parts in a given time, the "delirium", only differing in character; too much blood thrown with increased force, producing a fierce, active delirium, as in congestions of the meninges of the brain, whilst *too little* blood circulating with enfeebled force gives rise to a low, muttering, wandering sort of delirium, such as is witnessed in typhoid fever and typhoid pneumonia; the first to be controlled, moderated, brought down by depletants; the latter to be built up and equally benefitted by stimulants to be exhibited in full doses. I am sorry to say I have seen ice applied to the head in cases of typhoid diseases, when there was already *too little* blood circulating through the brain; and I always saw the case terminate fatally when this was done. In active delirium, no remedial agent is, in my opinion, better than bladders of ice; but in low, muttering delirium it is necessarily fatal, for it drives away from the brain the scanty supply of blood it is fed upon.

[TO BE CONTINUED.]

